# The *torso-like* gene functions to maintain the structure of the vitelline membrane in *Nasonia vitripennis,* implying its co-option into *Drosophila* axis formation

**DOI:** 10.1242/bio.046284

**Published:** 2019-09-05

**Authors:** Shannon E. Taylor, Jack Tuffery, Daniel Bakopoulos, Sharon Lequeux, Coral G. Warr, Travis K. Johnson, Peter K. Dearden

**Affiliations:** 1 Genomics Aotearoa and Biochemistry Department, University of Otago, P.O. Box 56, Dunedin, Aotearoa-New Zealand; 2 School of Biological Sciences, Monash University, 18 Innovation Walk, Clayton VIC 3800, Australia; 3 Otago Micro- and Nano- scale Imaging, University of Otago, PO Box 913, Dunedin, New Zealand, Aotearoa-New Zealand; 4 School of Medicine, University of Tasmania, 17 Liverpool St Hobart, TAS 7000, Australia

**Keywords:** Vitelline membrane, Terminal patterning, Axis formation, *Nasonia*, *Drosophila*, Evolution of development

## Abstract

Axis specification is a fundamental developmental process. Despite this, the mechanisms by which it is controlled across insect taxa are strikingly different. An excellent example of this is terminal patterning, which in Diptera such as *Drosophila melanogaster* occurs via the localized activation of the receptor tyrosine kinase Torso. In Hymenoptera, however, the same process appears to be achieved via localized mRNA*.* How these mechanisms evolved and what they evolved from remains largely unexplored. Here, we show that *torso-like*, known for its role in *Drosophila* terminal patterning, is instead required for the integrity of the vitelline membrane in the hymenopteran wasp *Nasonia vitripennis*. We find that other genes known to be involved in *Drosophila* terminal patterning, such as *torso* and *Ptth*, also do not function in *Nasonia* embryonic development. These findings extended to orthologues of *Drosophila* vitelline membrane proteins known to play a role in localizing Torso-like in *Drosophila*; in *Nasonia* these are instead required for dorso–ventral patterning, gastrulation and potentially terminal patterning. Our data underscore the importance of the vitelline membrane in insect development, and implies phenotypes caused by knockdown of *torso-like* must be interpreted in light of its function in the vitelline membrane. In addition, our data imply that the signalling components of the *Drosophila* terminal patterning systems were co-opted from roles in regulating moulting, and co-option into terminal patterning involved the evolution of a novel interaction with the vitelline membrane protein Torso-like.

This article has an associated First Person interview with the first author of the paper.

## Abstract (precis)

**Summary:** In the parasitic wasp *Nasonia*, Tsl, a key component of the process that defines the termini of the embryo of *Drosophila*, has a function in the structure of the vitelline membrane.

## INTRODUCTION

Establishment of the major axes and termini are some of the first events to take place during insect embryonic development. In *Drosophila*, the termini are patterned via the localized activation of the receptor tyrosine kinase Torso (Tor) by its ligand Trunk (Trk) which results in expression of the transcription factors Tailless (Tll) (Klingler et al., 1988; Strecker et al., 1989) and Huckebein (Bronner and Jackle, 1991, 1996) at the anterior and posterior of the embryo.

While localized activation of *tailless* is required at the termini, neither *trk* nor *torso* expression is localized (Casanova et al., 1995; Sprenger et al., 1989). Localization of signalling is provided by *torso-like* (*tsl*) (Savant-Bhonsale and Montell, 1993; Stevens et al., 1990), which encodes a protein present only at the termini of the embryo (Stevens et al., 2003). Tsl is expressed in subpopulations of follicle cells present at the anterior and posterior poles of the developing oocyte (Furriols et al., 2007; Stevens et al., 1990) and is secreted into the extracellular space between the follicle cells and oocyte where it associates with the vitelline membrane (VM) (Jiménez et al., 2002; Stevens et al., 2003). At the onset of embryogenesis, Tsl is proposed to transfer from the inner VM to the terminal plasma membrane of the embryo (Mineo et al., 2015; Stevens et al., 2003). Ectopic expression of *tsl* in all follicle cells causes the protein to be transported to the entire plasma membrane (Mineo et al., 2015) and loss of proteins that stabilize Tsl in the VM cause terminal pattering defects in addition to VM integrity problems (Ventura et al., 2010). The long-standing model is that Tsl localizes the activity of the Tor ligand (Trk) to the terminal regions, possibly via a mechanism involving the regulation of Trk secretion (Johnson et al., 2015), although other models have also been proposed (Amarnath et al., 2017; Mineo et al., 2018a,b).

The Torso pathway has other roles during development, such as in the initiation of metamorphosis (Rewitz et al., 2009). In this role, Tor responds to a different ligand, but one similar to Trk, prothoracicotropic hormone (PTTH). *Tsl* also has a role in regulating metamorphosis, however this is independent of Tor/PTTH signalling, and appears to be mediated via an effect on insulin signalling (Grillo et al., 2012; Henstridge et al., 2018; Johnson et al., 2013). Thus, the mechanism that controls Tor activation in the embryo is distinct from the one used to initiate metamorphosis. This implies that upstream factors involved in Tor activation (which we call the Tor activation cassette, or TAC) could be readily gained and lost during evolution.

Aside from these roles, *tsl* is also known to act in the development of the *Drosophila* immune system (Forbes-Beadle et al. 2016) and in morphogenesis (Johnson et al. 2017). In the latter, maternal *tsl* (but not *tor*) is required for formation of the ventral furrow during gastrulation.

Beyond *Drosophila*, the functions of *tsl*, and the other TAC genes, are unclear. In *Tribolium*, *tsl*, *tor* and *trk* are required for posterior terminal patterning (Schoppmeier and Schroder 2005, Grillo et al., 2012). Tsl protein and RNA are expressed at the termini of the oocyte (Schoppmeier and Schroder 2005; Baumer et al. 2012), and (like *Drosophila*) no localized expression of *tor* or *trk* has been reported. In hymenoptera (bees, wasps, ants and others), *trk* is missing from multiple sequenced genomes implying its loss early in hymenopteran evolution (Skelly et al., 2018). *PTTH* is missing from a more limited subset often correlating with loss of *tor*. Terminal patterning, via *tll* activation, instead relies on maternal localization of orthodenticle and/or tailless RNA (Lynch et al., 2006a,b; Wilson and Dearden, 2009, 2011). In the honeybee, where *PTTH*, *trk* and *tor* are absent (Dearden et al., 2006), maternal localization of posterior *tll* patterns the posterior terminal, while anteriorly expressed *otd* activates anterior *tll* expression (Wilson and Dearden, 2009). The parasitoid wasp, *Nasonia* (which lacks only *trk*), patterns its termini via localized maternal *otd* at both ends of the embryo (Lynch et al., 2006a,b). Since its genome contains a *tsl* gene, but one unlikely to be involved in terminal patterning, *Nasonia* provides an opportunity to explore conserved roles for *tsl* and may help us to understand how this gene has come to be involved in terminal patterning in *Drosophila*.

## RESULTS

### *Nasonia tsl* is expressed during oogenesis and in developing embryos

A *nasonia tsl* sequence was identified and its phylogenetic position assessed with respect to pan-arthropod Tsl proteins in (Skelly et al., 2018). We refer to this gene (NCBI LOC100118858) as *Nv-tsl*.

**Fig. 1.**
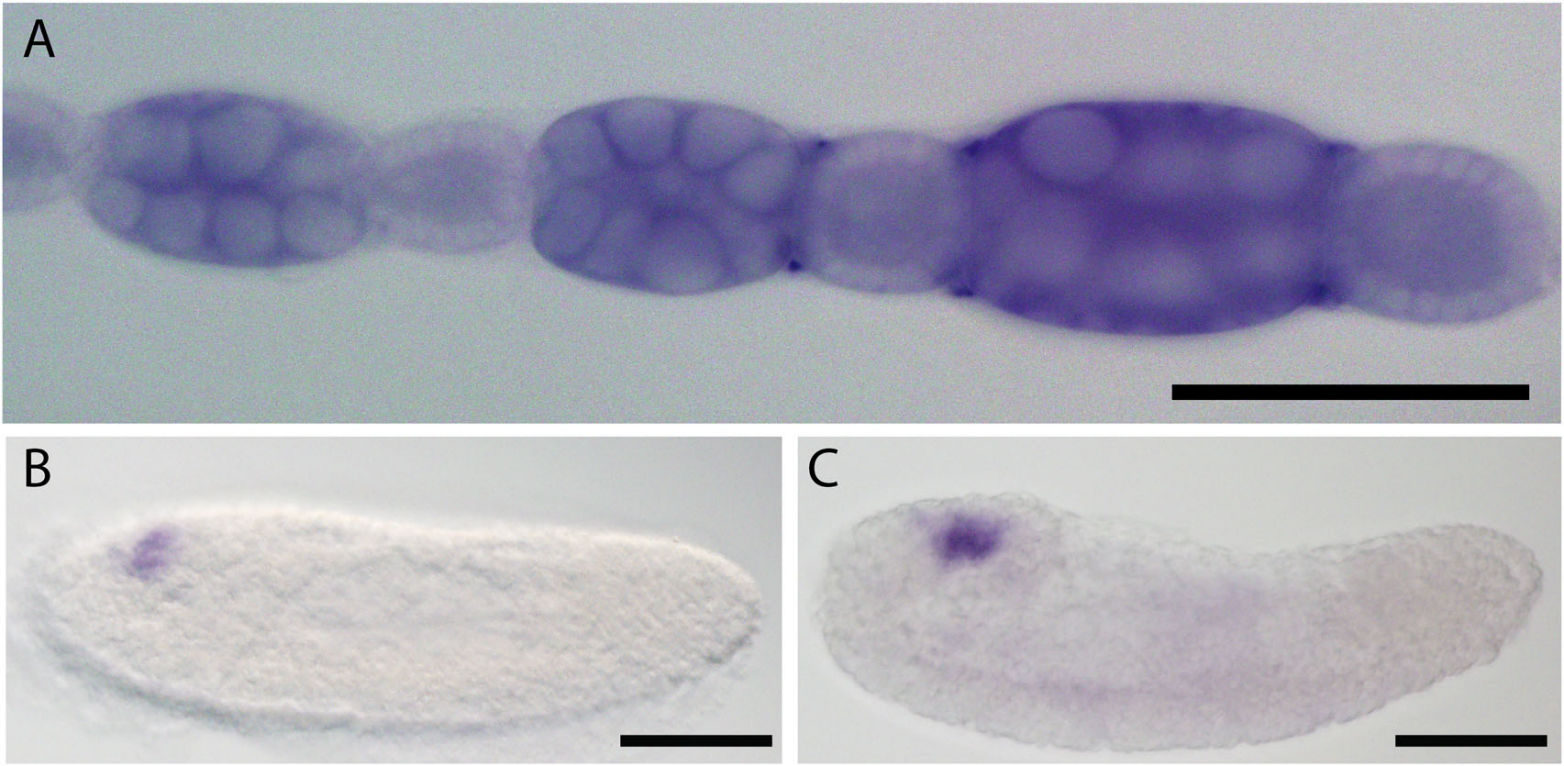
***Nv-tsl* expression in the *Nasonia* ovary and embryo** **as detected by *in-situ* hybridization.** (A) *Nv-tsl* RNA is present in all cell types of the *Nasonia* ovary. Staining is darker in nurse cells than the oocyte, consistent with maternal expression and RNA transport into oocytes. Dark spots at the outer boundary of oocyte and nurse cell clusters are an artifact due to probe trapping. (B,C) *Nv-tsl* is expressed in the embryonic brain in *Nasonia* embryos after germband extension. Negative controls shown in Fig. S3. Scale bars: 100 μm.

To determine if *Nv-tsl* is expressed in a pattern related to axis formation we investigated the expression of *Nv-tsl* RNA in *Nasonia* ovaries and embryos. In the ovary, *Nv-tsl* RNA is detected strongly in nurse cells, and weakly but ubiquitously in the oocyte and follicle cells, in a non-stage-specific manner (Fig. 1A). There is no specific localization of *Nv-tsl* RNA at the embryo termini, however we cannot rule out that protein is localized to the terminal regions. In the early embryo, *Nv-tsl* RNA is not expressed at the termini. Instead it is expressed in the embryonic brain during germband elongation (Fig. 1B,C). This expression pattern is consistent with *tsl* expression in the visual anlagen in *Drosophila* and near the cephalic lobe in aphids (Duncan et al., 2013) and may indicate a conserved role for *tsl* in brain function.

### Knockdown of *Nv-tsl* causes egg failure

**Fig. 2.**
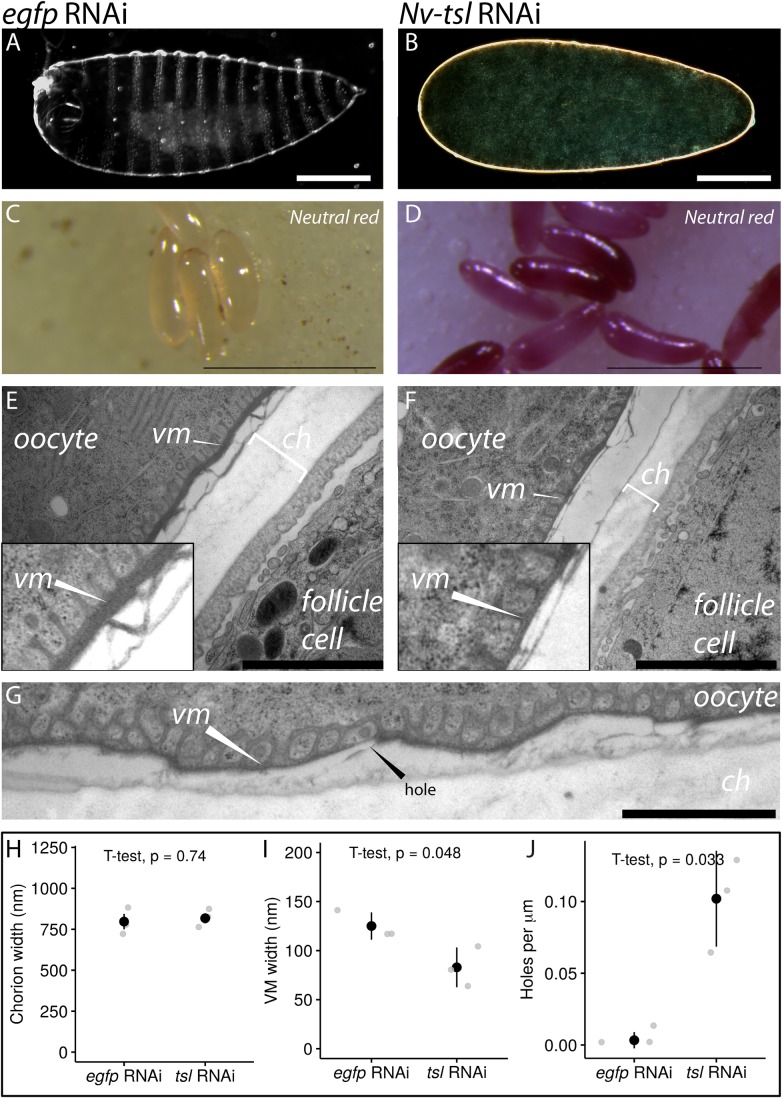
***Nv-tsl* is necessary for VM integrity in** ***Nasonia.*** (A,B) Cuticle preparations of *egfp* RNAi (A) and *Nv-tsl* RNAi (B) embryos. Scale bars: 100 μm. (C,D) Embryos from RNAi-injected females following Neutral Red assay. *Egfp* RNAi embryos (C) show no appreciable staining with Neutral Red, while *Nv-tsl* RNAi (D) embryos stain consistently. Scale bars: 500 µm. (E,F) Transmission electron micrographs of *egfp* RNAi (E) and *Nv-tsl* RNAi (F) ovaries. Scale bars: 2000 nm. ch, chorion (clear region); vm, vitelline membrane (electron dense region). The gap between the chorion and VM is due to the oocyte peeling away from the follicle cells. Insets show the location and thickness of the vitelline membrane in each treatment. (G) Transmission electron micrograph of *Nv-tsl* RNAi ovary showing thin chorion with a hole (marked). Scale bar: 1000 nm. (H) Quantification of chorion width for *egfp* and *Nv-tsl* RNAi-treated ovaries. For each image, the mean chorion width was measured. Data from 2­–11 images (technical replicates) were averaged for each biological replicate. There is no significant difference between chorion width between *egfp* and *Nv-tsl* RNAi-treated ovaries. (I) Quantification of VM width for *egfp* and *Nv-tsl* RNAi-treated ovaries. For each image, the mean VM width was measured. Data from 2–11 images (technical replicates) were averaged for each biological replicate. VM width differs significantly between *egfp* and *Nv-tsl* RNAi-treated ovaries. (J) Quantification of gaps in the VM for *egfp* and *Nv-tsl* RNAi-treated ovaries. The number of gaps per micron in each technical replicate was averaged to produce the mean number per biological replicate. *Egfp* RNAi-treated ovaries have significantly fewer gaps than those treated by *Nv-tsl* RNAi*.*

The maternal expression of *Nv-tsl* implies that it has a function in the ovary in *Nasonia*. To investigate this, we reduced *Nv*-*tsl* expression using maternal RNAi. This method has been used previously in *Nasonia* to investigate early axis formation (Lynch et al., 2006; Lynch and Desplan, 2006) and ovarian development (Lynch and Desplan 2006). Embryos collected from *Nv-tsl* RNAi-treated wasps (*Nv-tsl* knockdown) had a severe phenotype where many failed to form a cuticle, and thus embryonic structures, after 24 h (Fig. 2A,B). This implies that the embryos do not survive to form a cuticle, and reduction of *Nv-tsl* has an early, lethal effect on development. Surviving embryos were morphologically indistinguishable from wild type and are likely unaffected by RNAi, which is incompletely penetrant in *Nasonia* (Lynch and Desplan, 2006). Supporting this, *Nv-tsl* expression in *Nv-tsl* knockdown wasp ovaries was approximately half that of control wasps (see Fig. S1, *P*=0.0031, unpaired *t*-test, effect size=5.2, Cohen's d-test). Given the significant lethal effect of *Nv-tsl* parental RNAi, and the presumption that parental RNAi effects do not last all through embryogenesis (Rosenberg et al. 2014), we cannot determine in this experiment whether *Nv*-*tsl* acts in later developmental processes such as egg activation, terminal patterning or gastrulation. *Nv-tsl* may have roles in these processes that are masked by early lethality prior to the formation of cuticular pattern elements.

We infer that the lack of cuticle structure formation after 24 h indicates embryos were dying early in development. Indeed, some early (0–4 h old) embryos that we collected were flaccid to the touch. This phenotype is characteristic of a defect in one of the membranes surrounding the egg, the VM, a proteinaceous, heavily cross-linked matrix surrounding the oocyte (Waring, 2000). To further investigate this, we exposed embryos to Neutral Red, a vital dye used to reveal VM integrity problems in *Drosophila* (LeMosy and Hashimoto, 2000; Ventura et al., 2010; Waring, 2000) and eggshell integrity in *Aedes aegypti* (Isoe et al., 2019). Embryos with impaired eggshell integrity take up the dye and stain bright red. When *Nv-tsl* knockdown embryos were exposed to Neutral Red, 63% became stained, compared to 4.5% of embryos from control wasps injected with double-stranded RNA against the gene encoding enhanced green fluorescence protein (*egfp* RNAi) (Fig. 2C,D). This implies that *Nv-tsl* may be required for an aspect of eggshell biogenesis. Two membranes surround insect oocytes; outside the plasma membrane of the egg is the VM and beyond it lies the chorion. Since the chorions of embryos were not removed prior to Neutral Red staining, (as this is very difficult in *Nasonia*), our assay cannot distinguish between defects in the chorion and VM in *Nasonia*.

### *Nv-tsl* knockdown oocytes have defective VMs

As *Nv-tsl* RNAi embryos have early lethal phenotypes, we examined the ultrastructure of the ovary after RNAi against *Nv-tsl.* No morphological differences were detected between *Nv-tsl* knockdown and *egfp* knockdown using confocal microscopy after staining for actin and DNA (see Fig. S1). All key cell types; the follicle cells, nurse cells and oocytes, are indistinguishable from controls. In addition, there are no visible holes in the eggshell, and the oocyte nucleus is properly positioned. This implies that *Nv-tsl* does not interfere with gross ovary structure.

We therefore reasoned that the lack of visible eggshell defects might be due to insufficient resolution. To determine if *Nv-tsl* is required for VM or chorion integrity, we used high-pressure freezing and imaged *Nv-tsl* knockdown and *egfp* knockdown *Nasonia* ovaries using transmission electron microscopy.

Under electron microscopy, the chorion appears as a thick, electron-lucent (pale) layer. Comparing between *Nv-tsl* RNAi-treated and controls we found no significant differences in thickness of the chorion (Fig. 2G, *t*-test, *P*=0.72), nor were there any noticeable structural defects.

We next looked to see whether VM integrity might be compromised in *tsl* RNAi embryos.

The VM is a thin, electron-dense layer closely associated with the eggshell (Richards, 1969). Projections from the VM protrude towards the oocyte (Fig. 2E,F). We first noted that the VM of mature *Nv-tsl* RNAi oocytes appeared considerably thinner than that of controls. In addition, regions of the *Nv-tsl* knockdown VM contained holes that were rarely observed in control ovaries of equivalent stage (Fig. 2G).

To confirm this, we measured the VM thickness in micrographs where the VM appeared as a straight line, as we found a high degree of variability in regions where the VM curves around the oocyte surface. The average VM width of control ovaries was 125±35 nm, whereas the *Nv-tsl* knockdown VM was significantly thinner at 83±50 nm (*P*=0.048, effect size=−2.4, unpaired *t*-test and Cohen's d-test, *n*=3) (Fig. 2I). As another measure of VM integrity, we also counted the number of holes in the VMs of these wasp eggs (per micron of VM). We found that *Nv-tsl* knockdown eggs had significantly more VM holes than controls (*P*=0.033, unpaired *t*-test, effect size=4.1, Cohen's d test, *n*=3) (Fig. 2J). Taken together, our data imply that *Nv-tsl* knockdown in ovaries causes eggshell permeability via compromised VM integrity.

### Overexpression of *Nv-tsl* in *Drosophila* produces a spliced phenotype but does not rescue a *tsl* null mutation

**Fig. 3.**
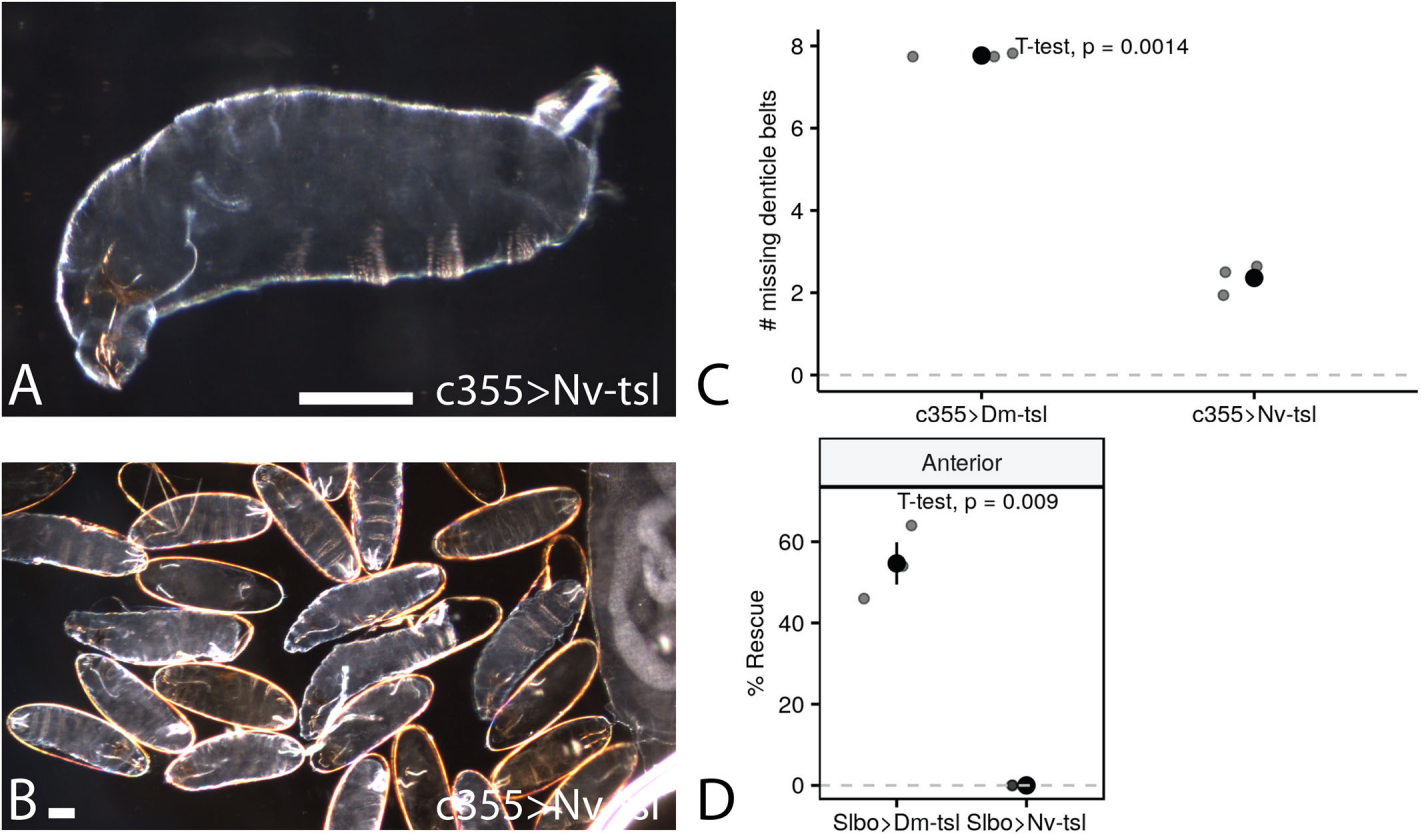
**Overexpression of *Nv-tsl* in *Drosophila* indicates** **some conservation of function.** (A) ‘Spliced’ *Drosophila* larva produced by overexpression of *Nv-tsl* using the c335 GAL4 driver, which expresses in *Drosophila* follicle cells. (B) Wide view of the results of larvae produced by overexpression of *Nv-tsl* using the c335 GAL4 driver to show range and variation of phenotypes. Scale bars: 100 μm. (C) Comparison of numbers of missing denticle belts from overexpression of *Drosophila tsl* and *Nv-tsl* using the c335 GAL4 driver. *Nv-tsl* expression causes the loss of fewer denticle belts than *Drosophila tsl.* (D) Rescue of a *tsl* deletion via expression of *Drosophila tsl* or *Nv-tsl* using the *Slbo* GAL4 line. *Drosophila tsl* rescues a deletion in the anterior of the embryos while *Nv-tsl* does not.

To determine if *Nv-tsl* has a different biochemical function to *Drosophila tsl,* consistent with its different biological effects when knocked down in *Nasonia*, we overexpressed *Nv-tsl* in *Drosophila* ovary tissue using the Gal4/UAS system (Brand et al., 1994) (Fig. 3A,B). When overexpressed from all follicle cells (via *c355*-Gal4) *Nv-tsl* causes loss of approximately two denticle belts on average per embryo (Fig. 3C), though to a lesser degree than *Dm-tsl* (loss of 7.8 denticle belts per embryo; Fig. 3C). We note that denticle belt loss is never observed in control embryos (*c355*-Gal4 alone), and that the denticle belt loss in both *Dm-tsl* and *Nv-tsl* overexpression embryos is statistically different to zero (*Dm-tsl*: *P*=2.40e-5; *Nv-tsl*: *P*=0.0162, one-sample *t*-test, *n*=3, mu=0, Bonferroni error correction). This denticle belt loss, known as a ‘spliced’ phenotype, results from expansion of the termini at the expense of central segments and is diagnostic of non-localized activation of terminal patterning (Klingler et al., 1988; Savant-Bhonsale and Montell, 1993). We further found that *Nv-tsl* is unable to rescue anterior terminal patterning in a *Drosophila tsl* null mutant background (Fig. 3D) when expressed using a strong anterior follicle cell driver (slbo-Gal4, *P*=0.18, one-sample *t*-test, *n*=3, mu=0, Bonferroni error correction). In this situation, *Dm-tsl* expression fully rescues more than half of the tested embryos (Fig. 3D, *P*=0.009, one-sample *t*-test, *n*=3, mu=0, Bonferroni error correction). This implies that *Nv-tsl* has a biochemical function similar to, but less effective than, *Dm-tsl* when placed in the *Dm* terminal system.

### *Nv-tsl* knockdown embryos do not phenocopy other putative VM proteins, which produce a contracted germband phenotype

In *Drosophila*, Tsl is localized to the VM by the *Drosophila* VM proteins encoded by *female sterile (1) Nasrat* [*fs(1)N*], *Female sterile (1) M3* [also called *Female sterile (1) polehole*) [*fs(1)M3*] and *Closca* (*Clos*), which stabilize the axis formation proteins Nudel and Tsl in the VM (Mineo et al., 2018a; Stevens et al., 2003; Ventura et al., 2010). To investigate potential terminal patterning functions of VM proteins we next determined the roles of *Nasonia* orthologues of *fs(1)N*, *fs(1)M3*, and *clos* in *Nasonia* embryonic development.

We identified *Nasonia* orthologues of *Drosophila fs(1)N*, *fs(1)M3*, *clos* using reciprocal blast*.* Phylograms of Maximum likelihood phylogeny inference (see Table S3 for alignment, and Fig. S2 for phylogram) indicate that LOC100169973 (XP_016843824) is the orthologue of *fs(1)N* [here named *Nv-fs(1)N*]; LOC103317810 (XP_008215462) is the *Nasonia* orthologue of *fs(1)M3* [here named *Nv-fs(1)M3*], and LOC100678101 (XP_016839710) is the *Nasonia* orthologue of *clos* (here named *Nv-clos*).

**Fig. 4.**
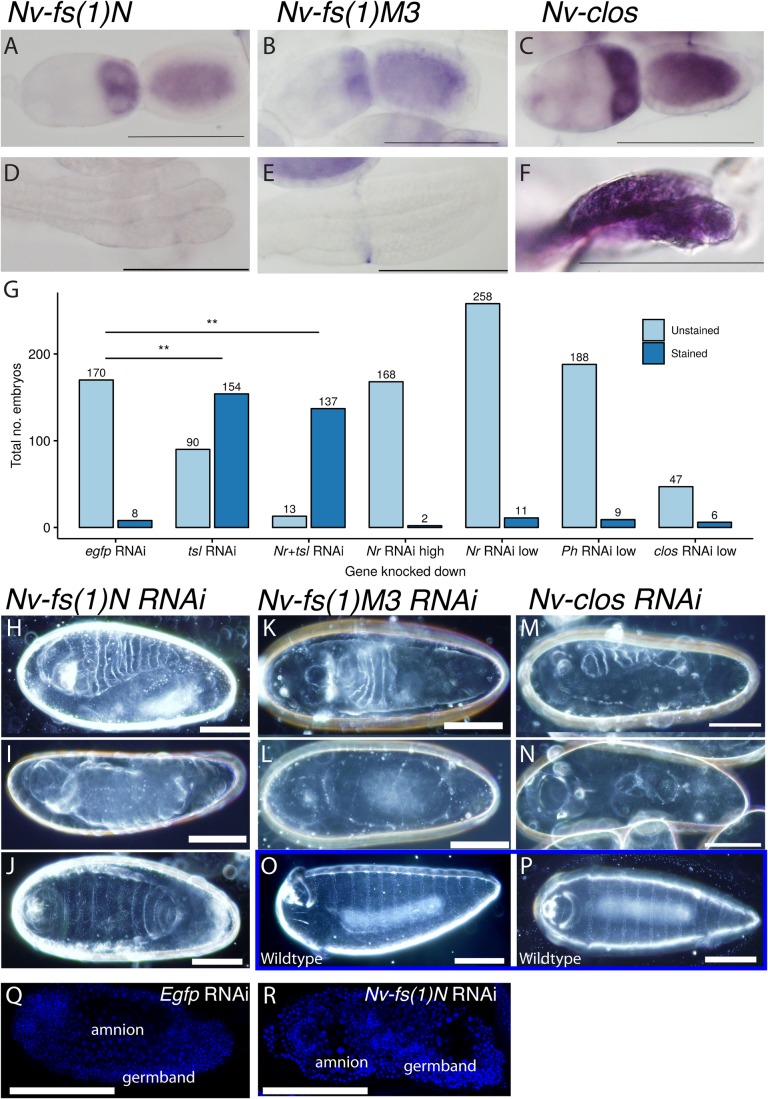
***Nv-fs(1)N*, *Nv-fs(1)M3* and *Nv-clos* are expressed in the ovary and cause embryonic defects when knocked down.** (A–F) *Nv-fs(1)N* (A,D), *Nv-fs(1)M3* (B,E) and *Nv-clos* (C,F) are provided maternally to oocytes as detected by *in situ* hybridization. *Nv-fs(1)N* (D) and *Nv-fs(1)M3* (E) expression is not detectable in the germarium of the ovary while *Nv-clos* (F) is. Negative controls shown in Fig. S3. (G) Quantification of embryos from females treated with various RNAi injections stained with Neutral Red to demonstrate integrity of embryo membranes. Gene names abbreviated for clarity. *Tsl*, *Nv-tsl*; *Nr*, *Nv-fs(1)N*; *Ph*, *Nv-fs(1) M3*; and *clos*, *Nv-clos. Nr+tsl* refers to injection with both *Nv-tsl* and *Nv-fs(1)N.* High RNAi refers to dsRNA concentrations ∼1000 ng/µl. Low RNAi refers to dsRNA concentrations 100–300 ng/µl. **P=1.596752e-32 between egfp RNAi and tsl RNAi; 5.560025e-54 between egfp RNAi and Nr+tsl RNAi. (H–N) Knockdown of *Nv-fs(1)N* (H–J), *Nv-fs(1)M3* (K,L) or *Nv-clos* (M,N) causes a range of phenotypes including contracted germband phenotypes (H,K,M) and apparent dorsal–ventral phenotypes (I,L,N). (J) ∼1% of embryos from females treated with *Nv-fs(1)N* RNAi exhibit loss of anterior and posterior segments. (O,P) Wild-type cuticles for comparison. (Q,R) Confocal section of DAPI-stained *Egfp* RNAi (O) or *Nv-fs(1)N* RNAi showing a twisted germband phenotype caused by maternal treatment of *Nv-fs(1)N* RNAi. Scale bars: 100 µm.

We examined the expression of *Nv-fs(1)N*, *Nv-fs(1)M3* and *Nv-clos* by *in situ* hybridization in the *Nasonia* embryos and ovaries, but were only able to detect expression in the latter. Here, *Nv-fs(1)N*, *Nv-fs(1)M3* and *Nv-clos* RNA are present in the oocyte and nurse cells, but are not detected in follicle cells (see Fig. 4A–C). *Nv*-*clos*, but not *Nv-fs(1)N* or *Nv-fs(1)M3*, is also detectable in the germarium (Fig. 4D–F). These data are consistent with the notion that *Nv-fs(1)Nr*, *Nv-fs(1)M3* and *Nv-clos* could encode structural constituents of the VM in *Nasonia*, as they do in *Drosophila*.

Using RNAi, we examined the impact of knocking-down expression of *Nv-fs(1)N*, *Nv-fs(1)M3* and *Nv-clos* maternally. We examined the possibility that *Nv-fs(1)N*, *Nv-fs(1)M3* and *Nv-clos* might be necessary for VM integrity in the same way that *Nv-tsl* is. Embryos from maternal RNAi for *Nv-fs(1)N*, *Nv-fs(1)M3* or *Nv-clos* are not permeable to Neutral Red (Fig. 4G), indicating their eggshells are intact.

Unlike the phenotype observed for *Nv-tsl* RNAi, these embryos developed through to late stages and it was possible to examine their cuticles. Maternal knockdown of *Nv-fs(1)N*, *Nv-fs(1)M3* or *Nv-clos* all cause severe embryonic defects (Fig. 4H–P). Knockdown of any one of these three genes appeared to cause embryonic defects of the germband (Fig. 4H,K,M), and possibly ventral specification (Fig. 4I,L,N). We also observed phenotypes similar to terminal defects in *Nv-fs(1)N* knockdown embryos (Fig. 4J). *Nv-fs(1)N* RNAi phenotype also causes the germband to be wrapped around the embryo on an angle, rather than oriented along the dorso–ventral axis (Fig. 4Q,R). None of these knockdown experiments give phenotypes similar to that of *Nv-tsl*, implying that these genes act in different processes. It is possible that *Nv-fs(1)N* has a function that influences terminal patterning but overall these data indicate that *Nv-fs(1)N*, *Nv-fs(1)M3* and *Nv-clos* may not be critical components of the VM in *Nasonia* as they are in *Drosophila*.

To investigate whether it is possible that the VM function of *Nv-Tsl* might be exacerbated or ameliorated by knockdown of orthologues of *Drosophila* VM genes, we performed double knockdown of *Nv-tsl* and *Nv-fs(1)N*. *Nv-tsl, Nv-fs(1)N* double-knockdown embryos were permeable to Neutral Red (Fig. 4G), indicating that these genes are unlikely to act in the same pathway.

### Other TAC components do not give terminal phenotypes, nor phenocopy *Nv-tsl* knockdowns

**Fig. 5.**
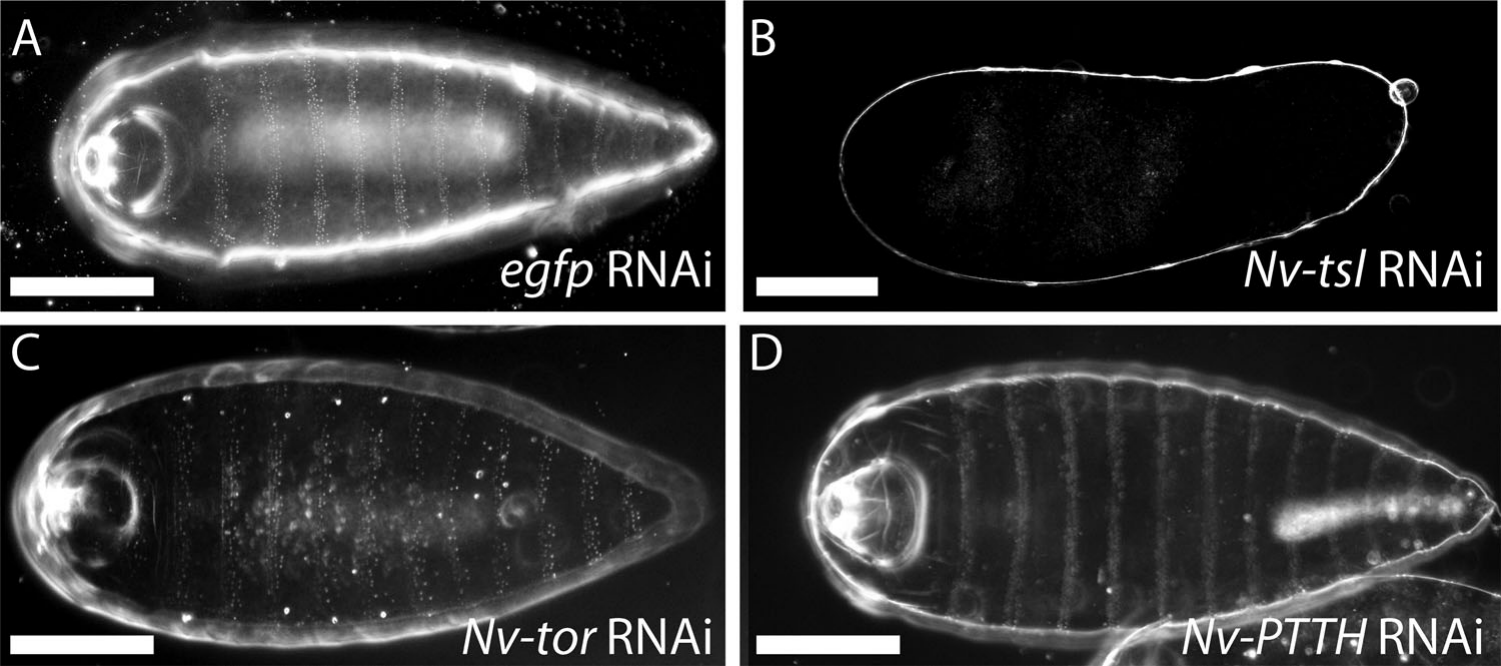
**Maternal RNAi knockdown of remaining** ***Nasonia* TAC components causes no embryonic phenotype.** (A) Example of a wild-type embryo from a female treated with *egfp* RNAi. (B) Example of an affected embryo from a female treated with *Nv-tsl* RNAi (representative of 100 embryos). (C,D) Example embryos from a *Nv-tor*-treated female (C, representative of 35 embryos), and a *Nv-PTTH*-treated female (D, representative of 41 embryos) both with wild-type patterns. Scale bars: 100 μm.

In *Nasonia*, *Nv-tsl* is necessary for VM integrity (Fig. 2). To investigate whether this function is coupled to the canonical terminal patterning system, we investigated the function of orthologues of the remaining TAC genes, *PTTH* and its receptor *torso* (previously identified by Skelly et al., 2018). Based on these data, we name LOC103315910 as *Nv-PTTH* and LOC103316970 as *Nv-torso.* Knockdown of either of *Nv-PTTH* or *Nv-torso* caused no overt cuticle phenotype (Fig. 5C,D), despite using very high concentrations of dsRNA (1000 ng/µl) in this experiment. Taken together, these data imply that *Nv-tor* and *Nv-PTTH* are not required for embryonic development in *Nasonia*, and that the TAC is neither involved in terminal patterning nor in mediating the VM function of *Nv-Tsl*.

## DISCUSSION

Here, we show that *Nv-tsl* is necessary for VM integrity in *Nasonia*. Functional evidence for this is twofold. (1) Embryos with reduced maternal *Nv-tsl* have permeable eggshells. (2) Electron microscopy reveals that the VM is thinner in *Nv-tsl* knockdown wasps compared to controls, potentially explaining the VM defect. Alongside this, *Drosophila* genes involved in VM function, *fs(1)N*, *fs(1)M3* and *clos*, cause embryonic patterning defects when knocked down in *Nasonia,* but not defects in the VM*.* Thus, the function of *Nv-tsl* in the VM is likely independent of *Nv-fs(1)N*, *Nv-fs(1)M3* and *Nv-clos*. Despite the difference in phenotype from reducing *Nv-tsl* in *Nasonia* compared to *tsl* mutants in *Drosophila*, the biochemical function of *Nv-tsl* is able to produce a ‘spliced’ phenotype when overexpressed in *Drosophila* follicle cells. This implies that while the biochemical function of Tsl may have remained similar over this evolutionary period, the consequences of that function have changed.

Our knockdown experiments involve maternal injection in *Nasonia*, which is thought to be unable to knock down zygotically acting developmental genes (Rosenberg et al. 2014). As the severe VM phenotype caused by *Nv-tsl* knockdown makes it impossible to detect other phenotypes of *Nv-tsl* knockdown, we cannot rule out a role for *Nv-tsl* in terminal patterning. Such a function seems unlikely, as all embryos collected from *Nv-tsl* knockdown mothers were either completely wild type or dead: there were no intermediate phenotypes from embryos with an intact VM but embryonic *Nv-tsl* knockdown. In addition, other *Nasonia* TAC genes do not act in *Nasonia* terminal patterning, and instead terminal patterning is known to be achieved via maternal localization of *otd* (Lynch et al., 2006a,b).

### Ancestral roles of *tsl*

The ancestral role of *tsl* is unclear. The gene is present throughout the Pancrustacea and is present in genomes lacking other TAC genes (Skelly et al. 2018). This implies it likely has an ancestral role independent from terminal patterning as carried out in *Drosophila* and from the other TAC genes. In support of this, the functional data presented here and from other studies imply that the ancestral role of *tsl* is not terminal patterning. In the most distantly related species to *Drosophila* examined, the hemipteran *Oncopeltus*, reduction of *tsl* expression causes germband invagination defects, but these appear to be caused by altered expression of *hunchback* and *giant*, but not *tll* or *huckbein* (Weisbrod et al. 2013).

Our data underscore the importance of the VM in early insect development. In *Drosophila*, both dorso–ventral and terminal axes are formed via proteins anchored in the VM. The terminal signal is provided by *tsl*, which is anchored in the VM by *Fs(1)N*, *Fs(1)M3* and *Clos* (Stevens et al., 2003). Nudel, required for the dorso–ventral axis, is also stabilized by these proteins in the VM (Mineo et al., 2017). The blastoderm embryos of both *Tribolium* and *Drosophila* are attached via integrins to the VM, ensuring the tissue expands asymmetrically during gastrulation (Münster et al., 2019).

The function of Tsl in the VM described here may be an ancestral one. The VM is laid down by the follicle cells during oogenesis (Cummings et al., 1971) and *tsl* is expressed in the follicle cells of every insect species surveyed to date, except the honeybee (Duncan et al., 2013). In addition, Tsl is known to be a component of the VM in *Drosophila*, the only other species where such evidence is available (Stevens et al. 2003). Therefore, *tsl* is plausibly present in the VM of most insects. Proposing this allows the reinterpretation for functional data from other taxa. Recent findings have implicated that the VM has an essential role in gastrulation (Münster et al., 2019), so it is plausible that a role of *tsl* in the structural integrity of the VM explains gastrulation phenotypes of *tsl* in *Drosophila* (Johnson et al. 2017) and *Oncopeltus* (Weisbrod et al. 2013).

If the ancestral function of *tsl* is in the insect VM, and it is present in the *Drosophila* VM, why do *Drosophila* not have the VM defects seen in *Nasonia* RNAi knockdown experiments? Our overexpression experiments imply that *Drosophila* and *Nasonia* Tsl share biochemical functions, so loss of *Drosophila* Tsl might be expected to cause VM holes. One possibility is that the change in function of Tsl from expression throughout the VM to just localization at the termini means that the knockdown phenotype is less severe in *Drosophila* and allows uncovering of the terminal (and other embryonic functions) activity of this gene.

### The evolution of canonical terminal patterning

**Fig. 6.**
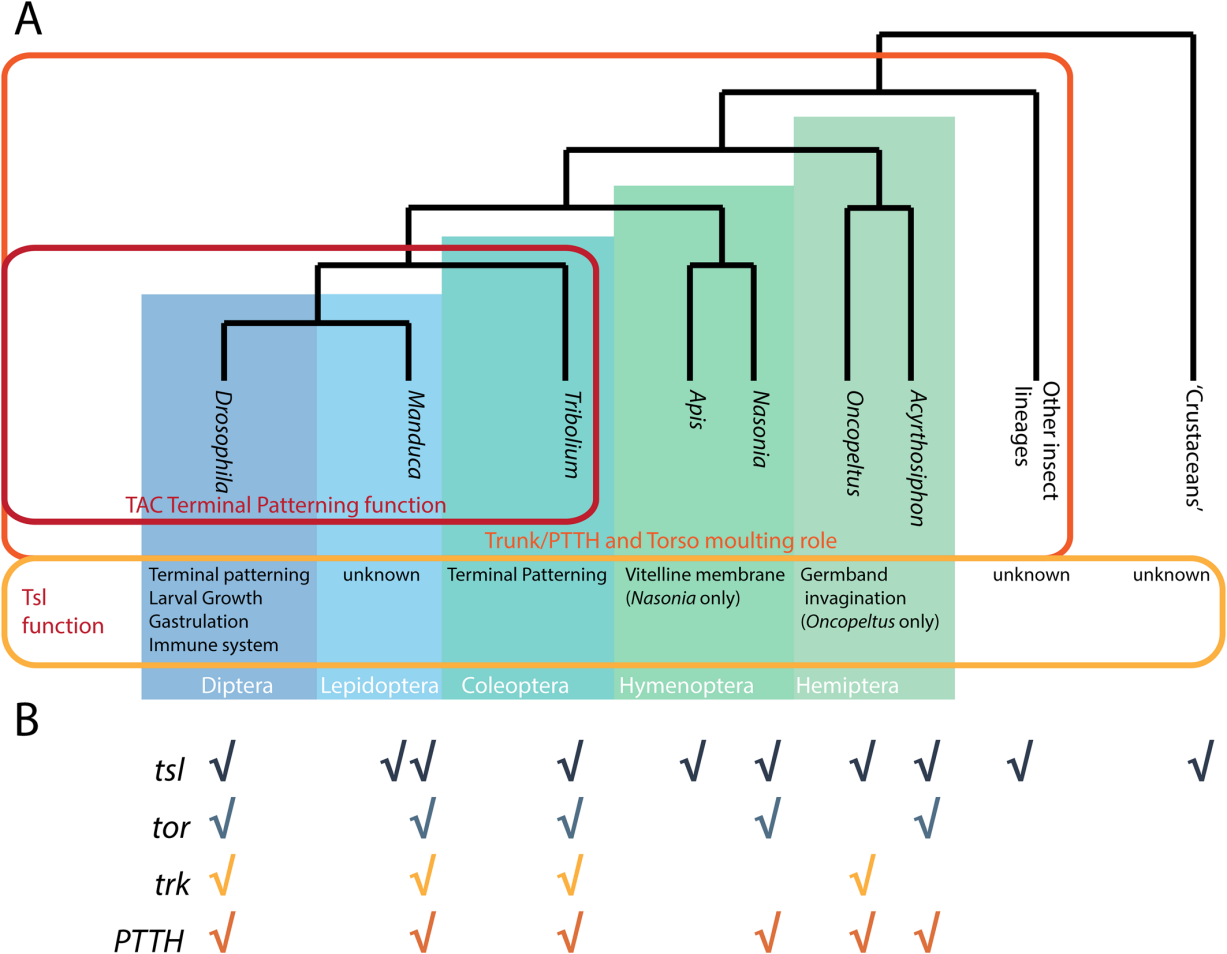
**Evolution of the Torso activation** **cassette.** (A) Phylogeny of arthropods with roles of TAC components, and particularly Tsl, marked. (B) Presence/absence of TAC components for the species listed in A, data from Skelly et al. (2018).

Our data support the hypothesis that the TAC genes were co-opted from other roles into terminal patterning (Duncan et al., 2013). Trunk/PTTH and Torso seem to have been co-opted from a more ancient role in moulting control (Duncan et al., 2013; Skelly et al., 2018). Our data from *Nasonia*, [a hymenopteran insect, the sister group to the rest of the holometabola (Krauss et al., 2004; Savard et al., 2006; Zdobnov and Bork, 2007)], implies an ancestral role of *tsl* could have been to ensure VM integrity (Fig. 6).

Co-option of Tsl into terminal patterning may be related to a change in the regulation of the *tsl* gene*,* and novel protein–protein interactions with Fs(1)N, Fs(1)M3, and Clos. In *Drosophila Fs(1)N* and *Fs(1)M3* mutants, the terminal expression domain of *tsl* in the VM is expanded, the total amount of *tsl* present is decreased, but localized expression is not completely lost (Stevens et al., 2003). Loss of *fs(1)N*, *fs(1)M3* and *clos* also reduces/abolishes Tsl in the VM of oocytes (Jiménez et al., 2002; Ventura et al., 2010). Thus, Fs(1)N, Fs(1)M3 and Clos-mediated localization of Tsl in the VM is necessary, but perhaps not sufficient, for Tsl stabilization in the VM.

The differences in terminal patterning between *Nasonia* and *Drosophila* provide some insight into the evolution of axis formation mechanisms. Both, unsurprisingly, rely on asymmetries set up maternally, either in the localization of some function of Tsl, allowing Torso activation in limited domains, or by localized placement of maternal RNA. It seems that the signals by which asymmetry is interpreted by the embryo to trigger axis formation are rapidly evolving and can involve many factors, including subtle regional differences in the VM.

The *Drosophila* terminal patterning system appears to have evolved through the coming together of two systems into the TAC. Our data provides a novel function for the only localized component of this system, *tsl*, in VM integrity. Existing hypotheses (Duncan et al., 2013) imply that the rest of the TAC, Tor*,* Trk and PTTH, are co-opted regulators of moulting. We propose the canonical terminal pathway evolved via co-option from two different processes (moulting and VM integrity/control) requiring novel interactions between moulting proteins and the ancestral VM protein Tsl*.*

## MATERIALS AND METHODS

### RNA interference and cuticle preparation

*Nasonia* were reared on *Lucilia sericata* blowflies (www.biosuppliers.com) at 25°C, after Werren and Loehlin (2009). For maternal RNAi, dsRNA was produced using run-off transcription from linearized plasmid (See Table S1 for plasmid sequences). This was carried out using the Invitrogen MEGAscript RNAi kit, per the manufacturer's instructions. Pupal microinjection and cuticle preparation were performed following Lynch and Desplan (2006) and Werren and Loehlin (2009). Concentrations of 150–500 ng/μl dsRNA were used for *Nv*-*tsl*, *Nv-fs(1) N* (*Nasrat*), *fs(1) ph* (*pole hole*) and *Nv*-*clos* (*closca*) knockdowns, and 1000 ng/μl for *Nv-torso* and *Nv-PTTH*.

### *In situ* hybridization

Dioxygenin-labelled probes for *in situ* hybridization were produced via run-off transcription. Ovaries for hybridization were dissected in PBS and placed into a 1:1 mix of 4% formaldehyde mix and heptane on ice, and fixed for 25 min before being stored in 100% methanol. Embryos were collected from vials with hosts placed into modified plugs (Lynch and Desplan, 2006). Hosts were cracked open and dipped into a 15 ml falcon tube containing 5 ml heptane, 4.5 ml PBS and 0.5 ml 37% formaldehyde (Invitrogen), and fixed for 8 h or overnight (Buchta et al., 2013). Ovary *in situ* hybridization was performed following Osborne and Dearden (2005) with ovaries digested for 12 min in proteinase K. Ovarioles were not separated before *in situ* hybridization. Embryo *in situ* hybridization was performed in the same manner, except embryos were digested for 5 min in 0.4 µl of proteinase K and hybridization was performed at 60°C.

### DAPI staining of embryos

Embryos were fixed as for *in situ* hybridization and stored in methanol without peeling. Embryos were then dehydrated through a 75%-50%-25% Methanol:PTw series for 5 min each, before being incubated with 1 µl/ml DAPI [4,6-Diamidino-2-Phenylindole, dihydrochloride (Invitrogen)] for 15 min, mounting in glycerol, and imaging under the FV1000 confocal microscope and 405 nm laser.

### *Tsl* overexpression and rescue in *Drosophila*

The following *Drosophila* stocks were used: *c355*-Gal4 (BL3750), *slbo*-Gal4 UAS-GFP (BL6458), *tsl^Δ^* and UAS-*tsl* (Johnson et al., 2013). To generate UAS-*Nv-tsl* transgenic lines, the open reading frame from *Nv-Tsl* (accession XM_001602685) was synthesized (Genscript) and ligated into pUASTattB via NotI/XbaI. Transgenic lines were generated by phiC31-mediated integration into the ZH-51C attP landing site (Bischof et al., 2007). Crosses were conducted at 25°C on standard fly media and adults were allowed to lay on media containing apple juice and agar supplemented with yeast paste at 25°C for 24 h before being removed. Embryos were developed for a further 24 h before dechorionation in 50% vol/vol bleach and mounting on slides in a mixture of 1:1 Hoyer's solution: lactic acid. Slides were incubated overnight at 65°C and imaged using dark field optics (Leica). Fifty individuals were scored per replicate and the data reported as the average number of denticle belts missing.

### qPCR

For qPCR, 1-day old wasp ovaries (∼8 days following RNAi) were dissected onto dry ice and stored at −80°C. RNA was extracted using the Qiagen RNeasy kit following the manufacturer's instructions. cDNA was produced using the Invitrogen SuperScriptIII kit. qPCR was performed after Duncan et al. (2013). The reference genes used were Rpn1 and L23 (NCBI LOC100115795, LOC100114985) and data was normalized to the mean Cq value of these genes. Data was analysed using R version 3.4.4 and the ‘pcr’ package version 1.1.2 (Ahmed 2018). See Table S2 for PCR primer sequences and efficiencies.

### Electron microscopy

*Nasonia* wasps were anaesthetized in fly vials on ice. Ovaries containing oocytes were dissected while submerged in ambient temperature PBS buffer and then transferred into Wohlwend #1093 copper gold-coated EMPACT membrane carriers (with a cavity diameter of 1450 µm and depth of 200 µm) and filled with 10% Ficoll (GE Healthcare 17-0310-10 Ficoll PM70) in 0.1 M Sorensen's phosphate buffer. Samples were frozen with a Leica EMPACT 2 High Pressure Freezer and stored in liquid nitrogen for 10 days.

Samples were transferred into a Leica EM AFS2 automatic freeze-substitution device (Leica Microsystems GmbH, Vienna, Austria). Tissue was stained with a freeze substitution medium consisting of 0.1% uranyl acetate, 1% glutaraldehyde and 1% osmium tetroxide (Electron Microscopy Science, Hatfield, USA) in acetone for 30 min at −130°C. Over 2 h the temperature was increased to −90°C. The freeze substitution medium was refreshed and held at −90°C for 44 h and 30 min. The temperature was then increased by 5°C per h until reaching −50°C where it was held for 8 h. The temperature was then increased by 10°C per h and held at −20°C for 2 h. Finally, the temperature was increased to 0°C over 1 h. The reagent baths containing the samples were removed from the AFS and all remaining infiltration steps were performed at room temperature.

Samples were infiltrated with a mixture of acetone with increasing concentrations of EMBED 812 epoxy resin [EMBED 812 Resin 14900 Electron Microscopy Sciences with medium hardness using accelerator BDMA (N-Benzyldimethylamine), 11400-25 Electron Microscopy Sciences] in the following series, 2:1, 1:1, 1:2 for 1 h each. Samples were then infiltrated with resin overnight with three resin changes. Blocks were polymerized for 48 h at 60°C.

Sections were cut on a Leica UC6 Ultramicrotome (Leica Microsystems, Germany) at ∼85 nm and mounted on formvar-coated copper slot grids (Electron Microscopy Science, Hatfield, USA). Grids were post-stained with uranyl acetate (Agar Scientific, Essex, UK) and lead citrate (cycle=UA, 20 min at 25°C, LC, 3 min at 25°C) with an LKB 2168 Ultrostain grid stainer (LKB-Produkter AB, Bromma, Sweden).

Sections were imaged with a Philips CM100 BioTWIN transmission electron microscope with LaB6 emitter (Philips/FEI Corporation, Eindhoven, The Netherlands) fitted with MegaView lll digital camera (Olympus Soft Imaging Solutions GmbH, Münster, Germany).

To calculate mean VM and chorion widths an ImageJ macro was used (originally http://imagej.1557.x6.nabble.com/Distance-Between-Lines-a-plugin-for-ImageJ-td3701802.html, also available on github https://github.com/Shannon-E-Taylor/tsl-project-scripts). In ImageJ, the two edges of the membrane being measured were traced manually, and a parallel line drawn between them. The macro then measured the distance between the traced lines, using the parallel line as a guide. A custom R script was used to calculate the mean membrane width for each image, and for each biological replicate (single ovary) (https://github.com/Shannon-E-Taylor/tsl-project-scripts).

### Neutral Red assay

0–4 h old embryos were collected into a ceramic staining dish in PTx (phosphate buffer solution+0.1% Triton 100), and washed three times in PTx. The PTx was then replaced with 5 mg/ml Neutral Red (Sigma-Aldrich) in PBS and incubated for 15 min at room temperature. Embryos were rinsed three times in PTx before being imaged.

## Supplementary Material

Supplementary information
