# Supplementary material for: The torso-like gene functions to maintain the structure of the vitelline membrane in Nasonia vitripennis, implying its co-option into Drosophila axis formation
Source: Biol Open. 2019 Sep 5;8(9):bio046284. doi: 10.1242/bio.046284 (PMC6777369; doi:10.1242/bio.046284)
Supplement: Supplementary information [file biolopen-8-046284-s1.pdf]

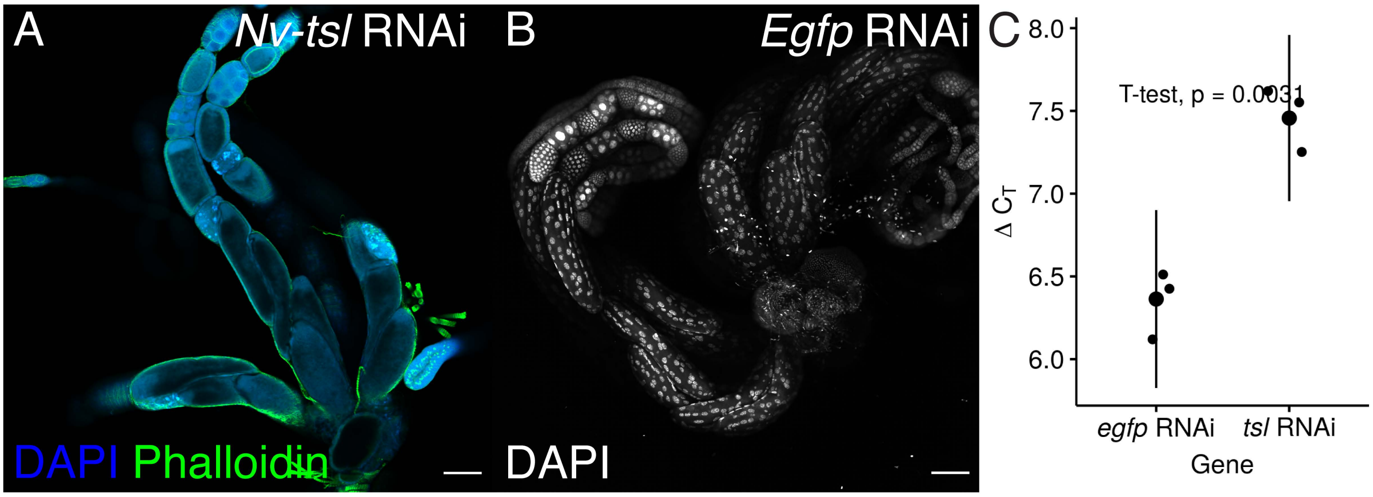

Figure S1: Nv-tsl RNAi knockdown in ovaries and qPCR.
A) Phalloidin and DAPI stained ovaries from *Nv-tsl* RNAi treated *Nasonia*
B) DAPI stained *Egfr*-RNAi treated ovary. Scale bars are 100 μm C) qPCR quantitation of *Nv-tsl* RNA in *Egfp* RNAi and *Nv-tsl* RNAi injected *Nasonia* expressed as ΔCT. *Nv-tsl* RNAi treated tissues have a higher ΔCT indicating significantly reduced amounts of *Nv-tsl* RNA.

Biology Open • Supplementary information

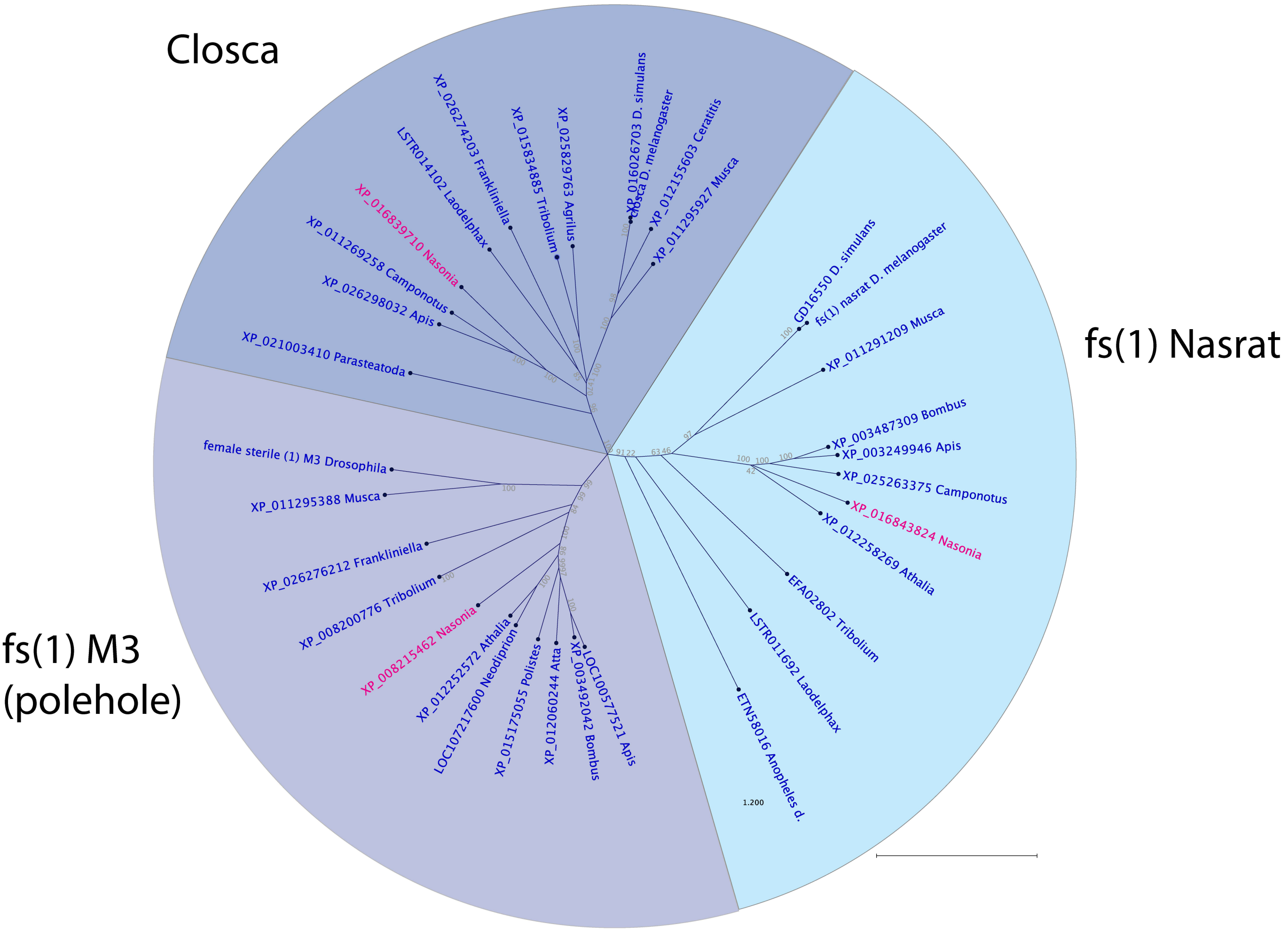

Figure S2: Phylogram of insect VM proteins. Proteins were identified in insect genomes as best reciprocal blast hits. Proteins were aligned with ClustalW (Thompson, Higgins et al. 1994) under standard settings and the Maximum Likelihood phylogenetic reconstruction carried out in CLC Main Workbench using the WAG model (Whelan and Goldman 2001). Bootstraps support percentage (from 1000 replicates) is shown at nodes.
Nasonia orthologues (in red) of *Drosophila* VM proteins are easily identifiable as either Closca, Fs(1)Nr or Fs(1)M3.

Thompson, J. D., D. G. Higgins and T. J. Gibson (1994). "CLUSTAL W: improving the sensitivity of progressive multiple sequence alignment through sequence weighting, positions-specific gap penalties and weight matrix choice." Nucleic Acids Research 22: 4673-4680.

Whelan, S. and N. Goldman (2001). "A general empirical model of protein evolution derived from multiple protein families using a maximum-likelihood approach." Mol Biol Evol 18(5): 691-699.

Biology Open • Supplementary information

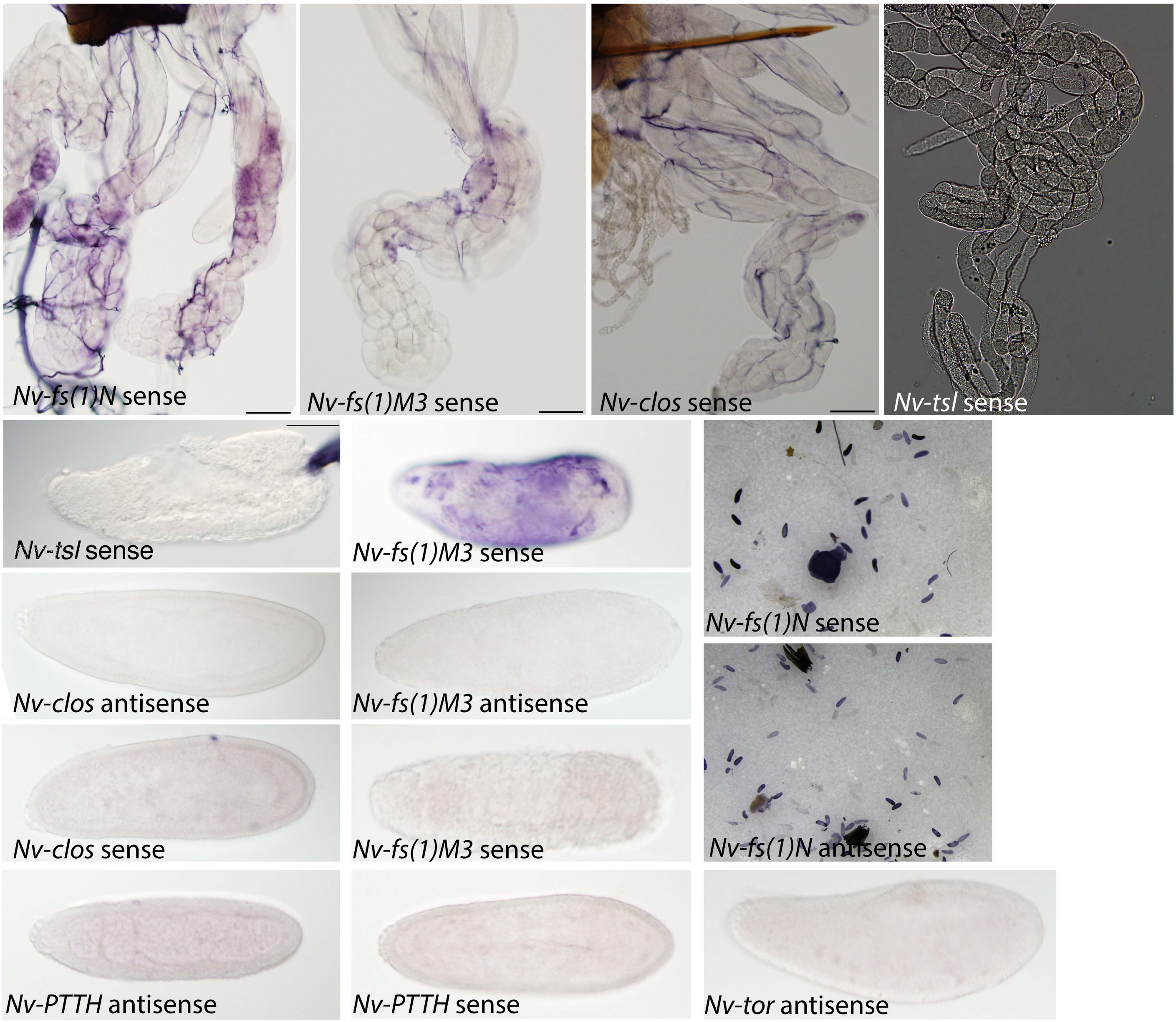

Figure S3: Sense (negative) controls for *in-situ* hybridistaion in ovaries and embryos. Antisense staining in embryos (showing no expression) for *Nv-fs(1)N, Nv-fs(1)M3, Nv-closca, Nv-PTTH* and *NV-tor.* Embryos are oriented with anterior to the left.

Biology Open • Supplementary information

Table S1: Sequences of clones used in this analysis.

Click here to Download Table S1

Table S2: PCR primers used in this analysis

Click here to Download Table S2

Table S3: ClustalW alignment of insect fs(1)N, fs(1)M3, and clos proteins

Click here to Download Table S3
